# Antibacterial, antioxidant and tyrosinase-inhibition activities of pomegranate fruit peel methanolic extract

**DOI:** 10.1186/1472-6882-12-200

**Published:** 2012-10-30

**Authors:** Olaniyi A Fawole, Nokwanda P Makunga, Umezuruike Linus Opara

**Affiliations:** 1 Postharvest Technology Research Laboratory, South African Research Chair in Postharvest Technology, Faculty of AgriSciences, Stellenbosch University, Private Bag X1, Stellenbosch, 7602, South Africa; 2 Department of Botany and Zoology, Faculty of Science, Stellenbosch University, Private Bag X1, Stellenbosch, 7602, South Africa

**Keywords:** Antibacterial activity, Tyrosinase-inhibition, Phenolics, Pomegranate, South Africa

## Abstract

**Background:**

This study evaluated, using *in vitro* assays, the antibacterial, antioxidant, and tyrosinase-inhibition activities of methanolic extracts from peels of seven commercially grown pomegranate cultivars.

**Methods:**

Antibacterial activity was tested on Gram-positive (*Bacillus subtilis* and *Staphylococcus aureus*) and Gram-negative bacteria (*Escherichia coli* and *Klebsiella pneumonia*) using a microdilution method. Several potential antioxidant activities, including radical-scavenging ability (RSA), ferrous ion chelating (FIC) and ferric ion reducing antioxidant power (FRAP), were evaluated. Tyrosinase enzyme inhibition was investigated against monophenolase (tyrosine) and diphenolase (DOPA), with arbutin and kojic acid as positive controls. Furthermore, phenolic contents including total flavonoid content (TFC), gallotannin content (GTC) and total anthocyanin content (TAC) were determined using colourimetric methods. HPLC-ESI/MS^n^ analysis of phenolic composition of methanolic extracts was also performed.

**Results:**

Methanolic peel extracts showed strong broad-spectrum activity against Gram-positive and Gram-negative bacteria, with the minimum inhibitory concentrations (MIC) ranging from 0.2 to 0.78 mg/ml. At the highest concentration tested (1000 μg/ml), radical scavenging activities were significantly higher in *Arakta* (83.54%), *Ganesh* (83.56%), and *Ruby* (83.34%) cultivars (P< 0.05). Dose dependent FIC and FRAP activities were exhibited by all the peel extracts. All extracts also exhibited high inhibition (>50%) against monophenolase and diphenolase activities at the highest screening concentration. The most active peel extract was the *Bhagwa* cultivar against monophenolase and the *Arakta* cultivar against diphenolase with IC_50_ values of 3.66 μg/ml and 15.88 μg/ml, respectively. High amounts of phenolic compounds were found in peel extracts with the highest and lowest total phenolic contents of 295.5 (*Ganesh*) and 179.3 mg/g dry extract (*Molla de Elche*), respectively. Catechin, epicatechin, ellagic acid and gallic acid were found in all cultivars, of which ellagic acid was the most abundant comprising of more than 50% of total phenolic compounds detected in each cultivar.

**Conclusions:**

The present study showed that the tested pomegranate peels exhibited strong antibacterial, antioxidant and tyrosinase-inhibition activities. These results suggest that pomegranate fruit peel could be exploited as a potential source of natural antimicrobial and antioxidant agents as well as tyrosinase inhibitors.

## Background

Numerous epidemiological studies suggest that diets rich in phytochemicals and antioxidants have protective roles in health and disease [1]. These natural antioxidants might play an important role in combating oxidative stress associated with degenerative diseases such as cancer, cardiovascular diseases, diabetes, Alzheimer’s disease and aging [2,3]. The antioxidative phytochemicals, especially phenolic compounds, found in vegetables and fruits have received increasing attention for their potential role in the prevention of human diseases [4-8].

Pomegranate (*Punica granatum* L.; Punicaceae) has gained popularity in recent years due to its multi-functionality and nutritional benefit in the human diet. The fruit is rich in tannins and other biochemicals, particularly phenolics, which have been reported to reduce disease risk [9,10]. Pomegranate fruit peel constitutes about 50% of the total fruit weight [11], and it is often discarded as waste. However, the fruit peel contains higher amounts of polyphenol compounds than the juice, and it possesses stronger biological activities [12-14]. Studies have shown that pomegranate peel extract had markedly higher antioxidant capacity than juice extract in scavenging against superoxide anion, hydroxyl and peroxyl radicals and it inhibited CuSO_4_-induced LDL oxidation [12]. Besides high antioxidant capacity, pomegranate peel extracts have been reported to possess a wide range of biological actions including anti-cancer activity [15-17], antimicrobial activity [18,19], anti-diarrheal activity [20], apoptotic and anti-genotoxic properties [21,22], anti-tyrosinase activity [23], anti-inflammatory and anti-diabetic activities [24,25]. Polyphenol compounds such as ellagic tannins, flavonols, anthocyanins, catechin, procyanidins, ellagic acid and gallic acid have been implicated in various pharmacological activities in the fruit peel [24-26]. However, the levels of these compounds in the pomegranate peel may vary among pomegranate cultivars which may result in differing levels of bioactivity [27].

In South Africa, more than ten pomegranate cultivars are being commercially cultivated [28]. Till date, there is no available information on bioactivities of fruit peels of pomegranate cultivars grown under South African agro-climatic conditions. If fruit peels of pomegranate cultivars show potential to improve human health, their utilisation should be encouraged during fruit processing. In the quest to promote the development of functional foods with health-benefiting properties, we investigated the antibacterial, antioxidant, and tyrosinase-inhibition activities of extracts from peels, using *in vitro* assays, of seven commercially pomegranate cultivars grown in the Western Cape, South Africa. Furthermore, the total phenolic content including flavonoid, gallotannin and anthocyanin content, and individual phenolics were quantified.

## Methods

### Plant material

The studies were performed on peels of seven pomegranate fruit cultivars (*Arakta*, *Bhagwa*, *Ganesh*, *Herskawitz*, *Molla de Elche*, *Ruby*, and *Wonderful*) which are commercially grown in South Africa. Fruit were procured from a commercial pomegranate pack house in Porterville (Western Cape Province). Fruit were harvested between February and May 2010, packed in paperboard cartons and transported in air-conditioned car to the Postharvest Research Laboratory. Immediately on arrival in the laboratory, ten fruits per cultivar were washed and manually peeled. The peels were freeze-dried, ground into powder form, and stored in airtight containers at 7°C in the dark.

### Preparation of peel extract

For each cultivar, each finely-powdered peel sample (2 g) was extracted separately with 10 ml of 80% (v/v) methanol (MeOH) and distilled water (aqueous) by sonication for 1 h [29]. The extract was filtered under vacuum through Whatman No.1 filter paper, and the residue was re-extracted further following the same procedure. Extracts were air-dried under a stream of air and first tested in the antibacterial assay to determine which extracts would be worth subjecting to further pharmacological investigations. Only the methanol extract was tested further in other assays, as it recorded highest antibacterial activity.

### Antibacterial property

#### Microdilution antibacterial assay

The antibacterial activity of peel extract was tested using the microdilution antibacterial assay for the minimum inhibitory concentration (MIC) values [30] as detailed by Fawole et al. [31], except that in the present study the initial concentration (50 mg/ml) of the sample was prepared by dissolving dried extracts in 80% (v/v) methanol. Two Gram-negative bacteria (*Escherichia coli* ATCC 11775 and *Klebsiella pneumonia* ATCC 13883) and two Gram-positive bacteria (*Bacillus subtilis* ATCC 6051 and *Staphylococcus aureus* ATCC 12600) were used. The extract was serially diluted two-folds with sterile distilled water in a 96-well micro-plate in triplicate for each of the four bacteria used. Streptomycin (0.1 mg/ml) was used as positive control, while water and bacteria-free broth were included as negative controls under the same conditions. Methanol (80%) was also included to check for false antibacterial activity. The final concentration of pomegranate extract ranged from 0.097 – 12.5 mg/ml, reducing the methanol content in the test extract to between 0.19 and 20%, whereas streptomycin was between 0.78 and 100 μg/ml.

### Antioxidant property

#### Radical-scavenging ability

The scavenging ability of stable free radicals such as 1,1-diphenyl-2-picrylhydrazyl (DPPH) is a known mechanism for antioxidation. The DPPH assay was carried out in triplicate, according to the method reported by Karioti et al. [32]. Extracts of different concentrations (10, 100 and 1000 μg/ml) were tested in triplicate for free-radical scavenging activity. The scavenging activity of the extract was compared with ascorbic acid (1000 μg/ml). A blank containing methanol instead of the test sample or ascorbic acid was also included under the same condition. The free radical scavenging activity (RSA) as determined by the decolouration of the DPPH solution was calculated according to the formula:

(1) $$\text{RSA}\;\left(\%\right)=\left[1–\left(A_{\text{test}}/A_{\text{blank}}\right)\;\text{x}\;100\right]$$

where *A*_test_ is the absorbance of the reaction mixture containing the standard antioxidant or extract, and *A*_blank_ is the absorbance of the blank test.

### Ferrous ion chelating (FIC) assay

The FIC activity assay of Singh and Rajini [33] was adopted. Briefly, 0.1 mM FeSO_4_ (0.5 ml) was mixed with the extract (0.5 ml) of different concentrations (10, 100 and 1000 μg/ml) in triplicate, followed by adding 0.25 mM ferrozine (1 ml). The reaction mixtures were incubated for 10 min and the absorbance (*A*) was measured at 562 nm. Ascorbic acid (1000 μg/ml) was included as the positive control. A blank test containing methanol instead of the test sample or ascorbic acid was also included under the same condition. The ability of extracts to chelate ferrous ions was calculated using the following equation:

(2) $$\text{Chelating ability}\;\left(\%\right)=\left[\left(A_{\text{blank}}–A_{\text{test}}\right)/A_{\text{blank}}\right]\;\text{x}\;100$$

where *A*_test_ is the absorbance of the reaction mixture containing extract or ascorbic acid and *A*_blank_ is the absorbance of the blank test.

#### Ferric ion reducing antioxidant power (FRAP) assay

The reducing power of extracts was measured according to the colourimetric method reported by Benzie and Straino [34] with a few modifications. In triplicates, methanolic extract (150 μl) of different concentrations at 10, 100 or 1000 μg/ml was added to 2850 μl of FRAP solution that constituted of 300 mM acetate buffer, 50 ml; 10 mM 2,4,6-tripyridyl-s-triazine (TPTZ), 5 ml; and 20 mM ferric chloride, 5 ml. Following the same procedure, a blank test containing 80% methanol instead of extract was included, while trolox at 10 μg/ml served as the positive control under the same condition. The reaction mixtures were incubated in the dark for 30 min. The reduction of the Fe^3+^-TPTZ complex to a coloured Fe^2+^-TPTZ complex by the extract was monitored by measuring the absorbance at 593 nm using a Helios Omega UV–vis spectrophotometer (Thermo Scientific technologies, Madison, USA). The changes in absorbance values of test reaction mixtures from the initial blank reading were considered as FRAP activity.

### Tyrosinase inhibition property

Tyrosinase inhibition activity was determined as described by Momtaz et al. [35], with _L_-3,4-dihydroxyphenylalanine (L-DOPA, Sigma) and tyrosine as substrates. Samples were dissolved in dimethyl sulfoxide (DMSO) to a concentration of 20 mg/ml, and further diluted in potassium phosphate buffer (50 mM, pH 6.5) to 600 μg/ml. Assays were carried out in a 96-well micro-titre plate and a Multiskan FC plate reader (Thermo scientific technologies, China) was used. All the steps in the assay were conducted at room temperature. In triplicate, each prepared sample (70 μl) was mixed with 30 μl of tyrosinase (333 Units/ml in phosphate buffer, pH 6.5). After 5 min incubation, 110 μl of substrate (2 mM _L_ -tyrosine or 12 mM L-DOPA) was added to the reaction mixtures and incubated further for 30 min. The final concentration of the extract was between 2.6 – 333.3 μg/ml. Arbutin (1.04 – 133.33 μg/ml) was used as a positive control while a blank test was used as each sample that had all the components except _L_-tyrosine or _L_-DOPA. Results were compared with a control consisting of DMSO instead of the test sample. Absorbance values of the wells were then determined at 492 nm. The percentage tyrosinase inhibition was calculated as follows:

(3) $$\%\;\text{inhibition}=\left[\left(A_{\text{control}}–A_{\text{sample}}\right)/A_{\text{control}}\right]\;\text{x}\;100$$

where *A*_control_ is the absorbance of DMSO and *A*_sample_ is the absorbance of the test reaction mixture containing extract or arbutin. The IC_50_ values of extracts and arbutin were calculated.

### Phenolic content determination

#### Total phenolic content (TPC)

The total phenolic (TP) content was determined in triplicate by the Folin-Ciocalteu (Folin-C.) colourimetric method [36] as modified by Makkar [37] and calculated as gallic acid equivalents (GAE) per gram DM.

#### Total flavonoid content (TFC)

Total flavonoid content (TFC) was determined using the method described by Yang et al. [38] and the results were expressed as catechin equivalent (CAE) per gram DM.

#### Rhodanine assay for gallotannin content (GTC)

Determination of the gallotannin content in peel methanolic extracts was carried out as described by Makkar [37]. Samples (50 μl) were mixed with 150 μl of 0.4 N sulphuric acid followed by 600 μl rhodanine. After 10 min, 200 μl of 0.5 N KOH were added and subsequently distilled water (4 ml) after 2.5 min. The absorbance was read at 520 nm (room temperature) against a blank test that contained aqueous methanol instead of the sample after 15 min incubation. The GTC was calculated from the standard curve (gallic acid) and expressed as gallic acid equivalents (GAE) per gram DM.

#### Total anthocyanin content (TAC)

Total anthocyanin content (TAC) was quantified using the pH differential method described by Wrolstad [39]. In triplicate, each extract (1 ml) was mixed with 9 ml of pH 1.0 and pH 4.5 buffers, in separate test tubes. Absorbance of the reaction mixture was measured at 520 and 700 nm in pH 1.0 and 4.5 buffers. The total absorbance was calculated from Equation 4, while total anthocyanin content was calculated from Equation 5. The result was expressed as cyanidin 3-glucoside.

(4) $$A={\left(A_{510}–A_{700}\right)}_{\text{pH}\;1.0}-{\left(A_{510}–A_{700}\right)}_{\text{pH}4}$$

(5) $$\text{Total anthocyanin}\;\left(\mu \text{g}/\text{ml}\right)=\left[\left(A\text{x MW x DF}\right)/\varepsilon \;\text{x L}\right]$$

*A* = Absorbance, ε = Cyd-3-glucoside molar absorbance (26,900), MW = anthocyanin molecular weight (449.2), DF = dilution factor, L = cell path-length (1 cm). Final results are expressed as Cyd-3-glucoside equivalent (C_3_gE) per gram dry matter (μg C_3_gE/g DM).

### HPLC-ESI/MS^n^ analysis of phenolic composition

The LC-MS analysis of phenolics and anthocyanin components in the pomegranate peel extract was performed according to Fischer et al. [40] with slight modification, using a Synapt G_2_ mass spectrometer UPLC^TM^ system (Waters Corp., Milford, USA) connected to a photo diode array detector and a BEH C18 column (1.7μm particle size, 2.1x100 mm, Waters Corp.). The mobile phases were 5% formic acid in water (v/v) as eluent A and 95% acetonitrile, 5% formic acid (v/v) as eluent B. The flow rate was fixed at 0.2ml/min and the column temperature was set at 40°C. The electrospray ionization (ESI) probe was operated in the positive mode with the capillary voltage of 3 kV; and cone voltage of 15 V. The injection volume was 10 μl and the detection was the diode array detector was set at between 200–600 nm. Individual phenolic compounds were quantified by comparison with a multipoint calibration curve obtained from the corresponding standard (catechin, epicatechin, protocatechuic acid, gallic acid, ellagic acid) from Sigma Aldrich (Germany), while anthocyanins were quantified by an external standard cyanidin 3, 5-diglucoside (Sigma Aldrich, Germany).

### Statistical analysis

All data are presented as mean values (±S.E). Analysis of variance (ANOVA) was performed using SPSS 10.0 for Windows (SPSS Inc. Chicago, USA). Where there was statistical significance (*P* < 0.05), the means were further separated using Duncan’s Multiple Range Test. Graphical analysis carried out using GraphPad Prism software version 4.03 (GraphPad Software, Inc. San Diego, USA). The IC_50_ values for the tyrosinase assay were calculated from the logarithmic non-linear regression curve derived from the plotted data using GraphPad Prism software version 4.03 (GraphPad Software, Inc., San Diego, USA).

## Results and discussion

### Antibacterial activity

Antibacterial activities of methanol and aqueous peel extracts of all the investigated pomegranate cultivars is presented in Table 1. None of the aqueous extracts exhibited good antibacterial activity at the highest screening concentration (> 12.5 mg/ml). The methanol extract, however, showed varying broad-spectrum antibacterial activity at statistically different MIC values (*P* < 0.05) against the test bacteria. Although it is ideal to test plant extracts against a wide range of target microorganisms, taxonomically representative bacterial species were used in this test to avoid handling numerous pathogenic microorganisms. The minimum inhibitory concentrations (MIC) were obtained for extract concentrations ranging from 0.78 to 0.20 mg/ml. In this study, MIC values less than 1.0 mg/ml were considered active for crude extracts [41]. Similar to the findings reported by Opara et al. [42] on peels of pomegranates grown in Oman, all peel extracts of the investigated fruit cultivars showed activity against the Gram negative and positive bacteria used. These findings are contrary to the work of Kanatt et al. [43], which reported that pomegranate extracts showed little or no effect with regards to Gram negative bacteria. The content of methanol used in the assay was inactive against tested bacteria in the assay. It is worth noting that although 80% methanol was used to dissolve the extracts, methanol concentration was <1.25% in all the extracts where the MIC values were record. The total antibacterial index (TAI) was calculated to determine the overall effects of the peel extracts of each cultivar of pomegranate studied against test bacteria. The most active cultivar was *Herskawitz* with the highest TAI value (6.25), while the lowest TAI value was exhibited by *Bhagwa* cultivar; clearly indicating that activity was cultivar dependent.

**Table 1:** Antibacterial activity of fruit peel methanol extracts of pomegranate cultivars cultivated in South Africa

		**Minimum inhibitory concentration (MIC; mg/ml)**				
**Cultivar**	**Extract**	**B.s**	**E.c**	**K.p**	**S.a**	**TAI**
Arakta	MeOH	0.39^b^	0.78^b^	0.20^a^	0.39^b^	6.00^b^
	Aqueous	>12.50	>12.50	>12.50	>12.50	<1.00
Bhagwa	MeOH	0.39^b^	0.78^b^	0.20^a^	0.78^c^	5.70^a^
	Aqueous	>12.50	>12.50	>12.50	>12.50	<1.00
Ganesh	MeOH	0.39^b^	0.39^a^	0.20^a^	0.78^c^	6.00^b^
	Aqueous	>12.50	>12.50	>12.50	>12.50	<1.00
Herskawitz	MeOH	0.20^a^	0.39^a^	0.20^a^	0.78^c^	6.25^c^
	Aqueous	>12.50	>12.50	>12.50	>12.50	<1.00
Molla de Elche	MeOH	0.39^b^	0.78^b^	0.39^b^	0.26^a^	5.92^ab^
	Aqueous	>12.50	>12.50	>12.50	>12.50	<1.00
Ruby	MeOH	0.39^b^	0.39^a^	0.39^b^	0.39^b^	6.00^b^
	Aqueous	>12.50	>12.50	>12.50	>12.50	<1.00
Wonderful	MeOH	0.39^b^	0.59^ab^	0.33^b^	0.39^b^	5.99^b^
	Aqueous	>12.50	>12.50	>12.50	>12.50	<1.00
Streptomycin (μg/ml)		3.13	3.13	2.60	5.21	

Mean values in the same column followed by different letters (a-c) represent statistical different (P <0.05) using the Duncan’s multiple range test*. MIC*- Minimum inhibitory concentration, *TAI* – Total antibacterial index, the higher the TAI the higher antibacterial activity.

Pomegranate peel polyphenols, especially tannins are the major components in the pomegranate peel extract that have been implicated in antimicrobial potential (for example, antiviral, antifungal and antibacterial activities) [44]. Vasconcelos [45] studied the antibacterial activity of methanolic peel extracts of pomegranate cultivars against both Gram negative and positive bacteria strains and reported MIC values ranging from 0.25 to 4.0 mg/ml against the test bacteria. The author reported a two-fold MIC value against a Gram positive bacterium (S*. aureus*) than against a Gram negative bacterium (*E. coli*). It has been suggested that the antimicrobial activity of tannins may be due to the ability of tannin compounds to precipitate proteins, therefore causing leakage of cell membrane of the microorganism [19], and aiding cell lysis which ultimately leads to cell death.

**Figure 1:**
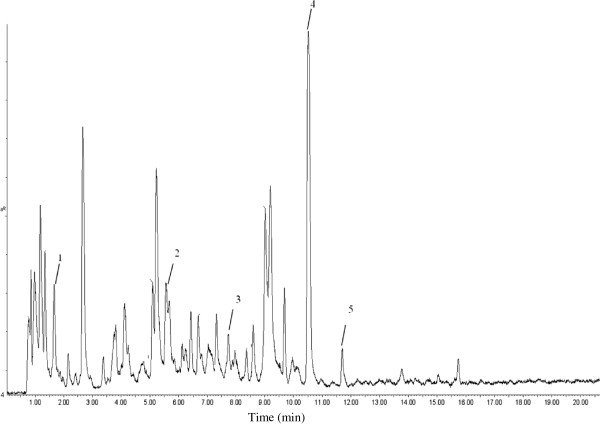
**Typical HPLC-MS chromatogram of methanolic peel extract of pomegranate fruit.** (1) Gallic acid; (2) Catechin; (3) Epicatechin; (4) Ellagic acid; (5) Rutin.

As the peel extracts are complex mixtures of metabolites (Figure 1), it is difficult to pinpoint all the metabolites that are responsible for pharmacological activity. Other researchers have postulated that punicalagin, along with a combination of various phytochemicals, plays a positive role in perceived bacterial inhibition. Considering the broad spectrum antibacterial property exhibited by the methanolic extracts, the fruit peel of the investigated pomegranate cultivars can be considered an effective antibacterial agent. Several reasons may explain varying antibacterial potency of the extracts tested here. Differences in antibacterial activities in the peel extracts may be linked to inter-genetic cultivar variability which is also associated with the inherent chemical composition of the fruit peel. Also, gene-to-biosynthetic modifications are well established in plants as they respond plastically to geo-environmental spatial variation [46]. Secondary metabolites play a key role as defence chemicals for many plants, and so, their accumulation is often dependent on environmental factors. Chemical heterogeneity ultimately leads to differences in the bioactivity of extracts derived from plants growing in different microclimatic areas as these plants face different abiotic and biotic challenges altering gene expression and secondary metabolite synthesis [47].

### Antioxidant activity

Negi and Jayaprakasha [48] extracted antioxidants from pomegranate peel with the use of methanol, acetone or water and found that methanol gave maximum antioxidant yield. In this study, some degree of radical scavenging activity (RSA) was observed in all the evaluated extracts, with considerable increase in RSA with increase in concentration level. Considering RSA of 50% as good activity, poor RSAs were exhibited by all the evaluated samples at concentrations of 100 μg/ml and 10 μg/ml (Table 2). However at the highest concentration tested (1000 μg/ml), the RSA was superior in all the fruit cultivars. The RSA values were significantly higher in *Arakta* (83.54%), *Ganesh* (83.56%), and *Ruby* (83.34%) cultivars (*p* < 0.05), while the lowest activity was exhibited by the *Molla de Elche* cultivar (71.65%). The RSA of ascorbic acid (67.02%), used in this study as a positive control, was lower than the plant extracts at 1000 μg/ml.

**Table 2:** Antioxidant activity of fruit peel methanol extracts of seven pomegranate cultivars cultivated in South Africa

	**DPPH (%)**			**FIC (%)**			**FRAP (abs. at 593 nm)**		
**Cultivar**	**1000 μg/ml**	**100 μg/ml**	**10 μg/ml**	**1000 μg/ml**	**100 μg/ml**	**10 μg/ml**	**1000 μg/ml**	**100 μg/ml**	**10 μg/ml**
Arakta	83.54±0.31^d^	13.35±0.98^ab^	5.55±0.06^c^	79.44±0.21^a^	49.94±0.89^a^	37.32±1.82^b^	1.19±0.03^ns^	0.52±0.02^b^	0.11±0.00^c^
Bhagwa	73.02±0.26^ab^	12.34±0.73^ab^	1.37±0.34^a^	84.96±1.43^bc^	65.54±1.09^c^	18.83±0.22^a^	1.03±0.28	0.38±0.00^ab^	0.04±0.01^a^
Ganesh	83.56±0.05^d^	16.70±0.83^bc^	2.42±0.99^ab^	82.98±0.18^b^	65.82±0.51^c^	15.80±0.52^a^	1.47±0.04	0.73±0.12^c^	0.08±0.01^bc^
Herskawitz	78.06±0.71^c^	15.18±0.97^abc^	2.71±0.77^ab^	87.82±0.57^de^	69.97±0.25^d^	34.32±2.45^b^	1.29±0.04	0.34±0.01^ab^	0.08±0.02^bc^
Molla de Elche	71.65±0.08^a^	10.59±0.18^a^	1.61±0.08^a^	86.59±0.90^cd^	70.57±0.43^d^	47.24±1.34^c^	1.47±0.11	0.33±0.05^a^	0.03±0.00^a^
Ruby	83.34±0.51^d^	19.67±2.24^c^	4.10±2.24^bc^	83.58±0.62^b^	53.39±1.29^b^	47.25±0.66^c^	1.18±0.02	0.38±0.00^ab^	0.08±0.01^bc^
Wonderful	74.19±1.05^b^	12.22±3.13^ab^	3.01±0.47^ab^	89.67±0.72^e^	71.02±0.38^d^	49.65±1.26^c^	1.32±0.16	0.33±0.01^a^	0.06±0.00^ab^
Ascorbic acid	67.02±0.06			62.15±0.98					
Trolox									0.82±0.03

Means in the same column followed by different letters (a-e) represent statistical significance (*P* <0.05) according to the Duncan’s multiple range test. Positive controls: Ascorbic acid and trolox.

The chelating ability of methanolic extracts of pomegranate peel on ferrous ion is presented in Table 2. Similar to the RSA results, the ferrous ion chelating (FIC) activity of methanolic peel extract exhibited a linear exponential relationship with extract concentration. Although low FIC activity was exhibited at the lowest concentration (10 μg/ml) assayed, extracts of *Molla de Elche*, *Ruby* and *Wonderful* showed relatively good FIC activity, ranging from 47.24 to 49.65%. At 100 μg/ml the FIC activity of all extracts (except *Arakta* cultivar) increased above 50%, with *Herskawitz*, *Molla de Elche* and *Wonderful*, showing highest values of 69.97%, 70.57% and 71.02%, respectively. Moreover, FIC activity exhibited by methanolic extracts of most of the investigated at 100 μg/ml were higher than that of the positive control (ascorbic acid at 1000 μg/ml). Dose dependent FIC activity exhibited by all the investigated extracts indicate that the pomegranate fruit peel contains constituents that inhibit oxidation through a mechanism other than radical scavenging activity.

The FRAP assay measures the ability of an antioxidant to reduce ferric (III) to ferrous (II) in a redox-linked colourimetric reaction that involves single electron transfer [12]. The reducing power of a compound serves as a significant indicator of its potential antioxidant activity. All the investigated extracts showed dose-dependent reducing power (Table 2). Interestingly, there was no significant difference (*p* < 0.05) in the reducing capacities among all the cultivars at the highest concentration (1000 μg/ml).

Antioxidant capacity based on both the free radical scavenging and the oxidation–reduction mechanisms may be determined by several methods, although the mechanism of action set in motion by antioxidant compounds is still not clearly understood [26]. Previous studies have shown strong antioxidant activity in pomegranate fruit peel extracts [12,13]. In comparison with other fruit peels, Okonogi et al. [49] studied the radical scavenging activity on DPPH and ABTS of pomegranate peel extract with other fruit types including rambutan, mangosteen, banana, coconut, dragon fruit, passion fruit as well as long-gong fruit. In the study the highest scavenging activity was reported in pomegranate peel extract. The observed antioxidant property in the peel extract in this study could be attributed to polyphenol compounds such as ellagic tannins, ellagic acid and gallic acid [24,50]. The results show that pomegranate peel may have great relevance in the prevention and therapies of diseases in which oxidants or free radicals are implicated, hence could serve as an economic source of natural antioxidants.

### Tyrosinase inhibition activity

**Figure 2:**
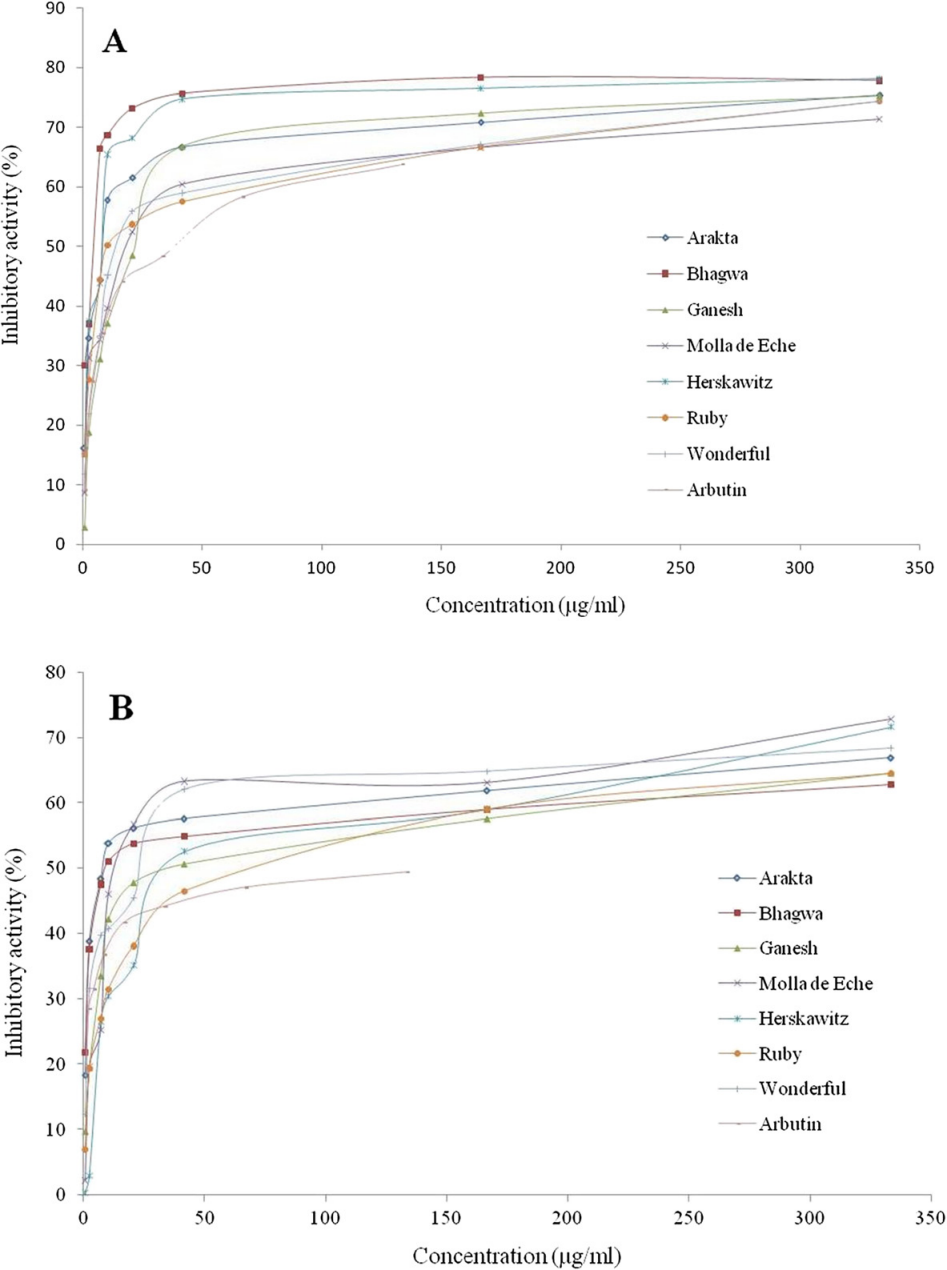
**Tyrosinase enzyme inhibitory activity of fruit peel methanol extracts of seven pomegranate cultivars cultivated in South Africa.** Monophenolase inhibition (**A**) and Diphenolase inhibition (**B**).

Tyrosinase inhibitors are chemical agents capable of reducing enzymatic reactions such as food browning and melanisation of human skin [23]. Results of tyrosinase inhibition activity of pomegranate methanol peel extract at different concentration (2.6–333.3 μg/ml) are presented in Figure 2 A and B against monophenolase (tyrosine) and diphenolase (DOPA), respectively, and activities were assessed in terms of dopachrome formation. Although the extracts were initially dissolved in 100% DMSO, the final content of the DMSO in the reaction mixture was between 0.14% and 18.3%. Also, DMSO was used as a control in the assay therefore the effect of DMSO (if any) would have been taken care of in the calculation. Generally, tyrosinase inhibition was displayed in a dose-dependent way and there was higher monophenolase inhibition than diphenolase inhibition. In this study, inhibition activity percentage above 50% was described as good tyrosinase inhibition. All extracts exhibited good inhibition against monophenolase and diphenolase activities at the highest screening concentration (Figure 2). Furthermore, there were significant (*p* < 0.05) differences in the concentrations of 50% tyrosinase inhibition (IC_50_) by the fruit peel. The most active peel extract was the *Bhagwa* cultivar against monophenolase and the *Arakta* cultivar against diphenolase with IC_50_ values of 3.66 μg/ml and 15.88 μg/ml, respectively (Table 3). The inhibitory activities of most of the peel extracts were higher than the positive control (arbutin) which is a known tyrosinase inhibitor. The IC_50_ values obtained in this study were higher than those reported by Yoshimura et al. [23] where IC_50_ value of 182.2 μg/ml was reported for 50% aqueous ethyl alcohol extract of pomegranate rind. Polyphenols are also the largest groups in tyrosinase inhibitors until now [51]. The pomegranate fruit peel is rich in polar substances such as polyphenol such as flavonoid constituents and tannins. In solution, the defining characteristic of tannins is the ability to precipitate mainly proteins, and the structure of flavonoid is compatible with the roles of both substrates and inhibitors of tyrosinase [51]. It could be suggested that tannin content in pomegranate peel could precipitate tyrosinase enzyme, thereby inhibiting enzymatic activity in the reaction medium. These constituents are readily soluble in methanol and show high tyrosinase inhibitory activity in different plants [52,53].

**Table 3:** **Effective inhibition concentration (EC**_**50**_**) of fruit peel methanol extracts against tyrosinase**

**Cultivar**	**IC**_**50**_**Monophenolase (μg/ml)**	**IC**_**50**_**Diphenolase (μg/ml)**
Arakta	11.03±0.08^c^	15.88±0.10^a^
Bhagwa	3.66±0.11^a^	21.16±0.09^a^
Ganesh	25.38±0.06^f^	40.93±0.12^b^
Herskawitz	7.56±0.08^b^	59.03±0.07^c^
Molla de Elche	25.56±0.06^f^	27.11±0.09^ab^
Ruby	20.33±0.07^d^	114.9±0.08^e^
Wonderful	23.67±0.06^e^	27.26±0.07^ab^
Arbutin	34.66±0.05^g^	98.66±0.12^d^

Mean values in the same column followed by different letters (a-f) represent statistical different (P <0.05) using the Duncan’s multiple range test.

According to Yoshimura et al. [23], ellagic acid in pomegranate rind showed an inhibitory effect on tyrosinase *in vitro* and a whitening effect *in vivo* on UV-induced pigmentation of brownish guinea pig skin. On the contrary, however, Chang [51] argued that some phenolic compounds could be mistakenly classified as tyrosinase inhibitors due to their role as alternative enzyme substrates whose quinoid reaction products absorb in a spectral range different from that of dopachrome. As a result, when the phenolics show a good affinity for the enzyme, dopachrome formation is prevented.

### Phenolic compounds analysis

#### Total phenolics, flavonoid, gallotannin and anthocyanin

Pomegranate fruit components are rich in phenolic compounds which have synergistic and/or additive effects on its pharmacological properties [22]. Phenolic constituents in pomegranate peel have been implicated in bioactivities such as antimicrobial, antioxidant, and anti-tyrosinase activities [23,24,44]. Results obtained in this study revealed significant cultivar differences (*p* < 0.05) in the levels of phenolic compounds (Table 4). The *Ganesh* cultivar had the highest amount of total phenolics (295.5 mg/g DM) whereas *Molla de Elche* cultivar had the lowest phenolic contents (179.3 mg/g DM). These results corroborate the levels of total phenolic content (249.4 mg/g) reported by Li et al. [12], who also found that total phenolic content of pomegranate peel extract was 10-fold as high as that of the juice extract. Similarly, the highest (126 mg/g DM) and lowest (97.8mg/g DM) contents of total flavonoid were measured in *Ruby* and *Wonderful* cultivars, respectively. This result was expected as flavonoid is a major phenolic group in pomegranate and should contribute to the total phenolic content in the peel extracts. Flavonoid contents found in the investigated cultivars were higher than the value reported by Li et al. [12]. Although the gallotannin content in *Arakta* (783.6 μg/g DM) was insignificantly higher than that of *Ganesh* (777.2 μg/g DM) the cultivar contained higher amount of gallotannin than in other cultivars. Anthocyanin is one of the most important groups of flavonoid which is responsible for red colouration of pomegranate fruit [54]. Total anthocyanin was the highest in *Wonderful* (322.2 μg/g DM) whereas the lowest was contained in the *Molla de Elche* cultivar (58.5 μg/g DM).

**Table 4:** Phenolic contents in fruit peel methanol extracts of seven pomegranate cultivars cultivated in South Africa

**Cultivar**	**Total phenolics**	**Total flavonoid**	**Total gallotannin**	**Total anthocyanin**
	**mg GAE/ g DM**	**mg CAE /g DM**	**μg GAE/ g DM**	**μg C**_**3**_**gE /g DM**
Arakta	187.4±6.44^ab^	103.0±1.86^a^	783.6±65.11^d^	289.7±1.63^d^
Bhagwa	224.1±6.86^c^	112.6±1.51^b^	697.7±42.92^cd^	312.6±1.25^e^
Ganesh	295.5±23.91^d^	121.1±3.12^c^	777.2±34.28^d^	65.1±1.00^a^
Herskawitz	198.1±9.22^abc^	101.0±1.02^a^	530.1±33.86^b^	195.9±2.25^c^
Molla de Elche	179.3±4.60^a^	99.5±2.94^a^	560.3±62.08^bc^	58.5±1.27^a^
Ruby	218.2±4.53^bc^	126.0±0.57^c^	326.0±35.28^a^	111.7±3.51^b^
Wonderful	189.1±3.79^ab^	97.8±2.10^a^	466.3±69.4^ab^	322.2±11.90^f^

*GAE*, gallic acid equivalent; *CAE*, catechin equivalent; *C*_*3*_*gE*, cyanidin-3glucoside equivalent; *DM*, dry mass. Mean values in the same column followed by different letters (a-c) represent statistical different (P <0.05) using the Duncan’s multiple range test.

### HPLC-MS^n^ analysis of phenolic composition

**Figure 3:**
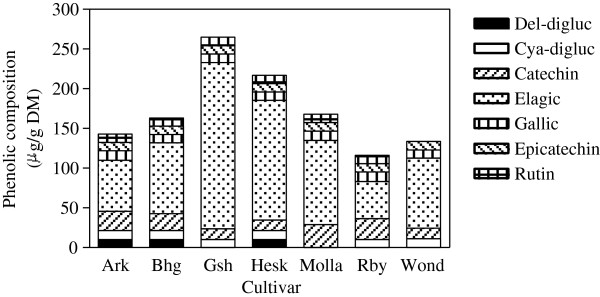
**Phenolics composition in peel methanol extracts of seven pomegranate cultivars.** Ark- *Arakta*, Bhg- *Bhagwa*, Gsh- *Ganesh*, Hesk- *Herskawitz*, Molla- *Molla de Elche*, Rby- *Ruby* & Wond- *Wonderful*.

Individual phenolic compounds in pomegranate fruit peel such as punicalagin, ellagic acid, gallic acid, caffeic acid, protocatechuic acid and p-coumaric acid have received considerable attention due to their potent antibacterial, antioxidant and anti-tyrosinase activities [22,24,44,50,55,56]. High-performance liquid chromatography-mass spectrometry (HPLC-MS) was used to determine the individual concentrations of the most prominent phenolic compounds in the methanolic peel extract of pomegranate cultivars studied. As shown in Figure 3, seven individual phenolics were identified and quantified, namely; anthocyanins: delphinidin 3,5-diglucoside, cyanidin 3,5-diglucoside flavonoids: catechin, epicatechin and rutin; hydrolysable tannin: ellagic acid; and hydroxybenzoic acid: gallic acid. Phenolic profile and concentration varied amongst the fruit cultivars. Catechin, epicatechin, ellagic acid and gallic acid were found in all cultivars, of which ellagic acid was the most abundant comprising of more than 50% of total phenolic compounds detected in each cultivar. The concentration of ellagic acid ranged from 46.87 μg/ml (*Ruby*) to 209.44 μg/ml (*Ganesh*). The anthocyanin types; delphinidin 3,5- diglucoside and cyanidin 3,5- diglucoside, were detected in *Arakta*, *Bhagwa* and *Herskawitz* cultivars, while *Ganesh*, *Ruby* and *Wonderful* cultivars contained only cyanidin 3,5- diglucoside (Figure 3).

Noda et al. [57] reported that cyanidin, pelargonidin, and delphinidin were the principal anthocyanins in pomegranate peel. In the present study, Rutin was found in all cultivars except in *Wonderful* cultivar, and catechin was the highest in *Molla de Elche* cultivar with a concentration of 28.85 μg/ml. Overall, the total concentration of the identified phenolic compounds was in the order of *Ganesh* >*Herskawitz* >*Molla de Elche* >*Bhagwa* >*Arakta* >*Wonderful* >*Ruby*. The presence of these polyphenols in the pomegranate peel may be responsible for the bioactivities observed in the methanol extracts. Phenolic types contained in plants influence antimicrobial activity of the plants [58]. For instance, flavone, quercetin and naringenin were reported showing strong inhibitory activity on the growth of *Aspergillus niger*, *Bacillus subtilis*, *Candida albicans*, *Escherichia coli*, *Micrococcus luteus*, *Pseudomonas aeruginosa*, *Saccharomyces cerevisiae*, *Staphylococcus aureus* and *Staphylococcus epidermidis*, while gallic acid inhibited only *P. Aeruginosa* whereas no inhibitory activity was exhibited by rutin and catechin on the tested microorganisms [58]. Major chemicals identified through LCMS may not be the only compounds responsible for bioactivity in the pomegranate peel extracts. Other compounds not identified may play a more significant role in the biological activities exhibited by the peel extracts.

## Conclusion

This study has shown that the peel of the investigated pomegranate fruit cultivars possess strong antibacterial, antioxidant and anti-tyrosinase activities. Therefore the peel of the pomegranate fruit cultivars, instead of being wasted, could be exploited as a potential source of natural antimicrobial and antioxidant agents, as well as a potential tyrosinase inhibitor. The findings provide scientific basis to promote value-adding of pomegranate fruit peels for pharmaceutical and cosmetic purposes. Further studies on the isolation of active ingredients, determination of cytotoxicity and genotoxicity effects as well as the mode of action of tyrosinase-inhibitory, antibacterial and antioxidant properties in pomegranate peel extracts are warranted.

## Competing interests

The authors declare that they have no competing interests.

## Authors' contributions

OAF was involved in sample collection, carried out the antibacterial, antioxidant and tyrosinase assays as well as statistical analysis, and also drafted the manuscript. NPM was heavily involved in the antibacterial assay as well as phytochemical and HPLC-MS analyses, and was also mainly involved in scientific correction of the draft manuscript. ULO designed and supervised the study, and revised the manuscript for critically important content. All authors approved the final manuscript.

## Pre-publication history

The pre-publication history for this paper can be accessed here:

http://www.biomedcentral.com/1472-6882/12/200/prepub
